# Diazoxide is a powerful cardioprotectant but is not feasible in a realistic infarct scenario

**DOI:** 10.3389/fcvm.2023.1173462

**Published:** 2023-04-19

**Authors:** Petra Kleinbongard, Helmut Lieder, Andreas Skyschally, Gerd Heusch

**Affiliations:** Institute for Pathophysiology, West German Heart and Vascular Center, University of Duisburg-Essen, Essen, Germany

**Keywords:** cardioprotection, coronary microvascular obstruction, diazoxide, infarct size, myocardial infarction, reperfusion

## Abstract

**Introduction:**

Diazoxide is a powerful cardioprotective agent that activates mitochondrial ATP-dependent K-channels and stimulates mitochondrial respiration. Diazoxide reduced infarct size in isolated rodent heart preparations and upon pretreatment in juvenile pigs with coronary occlusion/reperfusion. We aimed to study the use of diazoxide in a more realistic adult pig model of reperfused acute myocardial infarction when diazoxide was administered just before reperfusion.

**Methods and results:**

In a first approach, we pretreated anaesthetised adult Göttingen minipigs with 7 mg kg^−1^ diazoxide (*n* = 5) or placebo (*n* = 5) intravenously over 10 min and subjected them to 60 min coronary occlusion and 180 min reperfusion; blood pressure was maintained by use of an aortic snare. The primary endpoint was infarct size (triphenyl tetrazolium chloride staining) as a fraction of area at risk; no-reflow area (thioflavin-S staining) was the secondary endpoint. In a second approach, diazoxide (*n* = 5) was given from 50 to 60 min coronary occlusion, and blood pressure was not maintained. There was a significant reduction in infarct size (22% ± 11% of area at risk with diazoxide pretreatment vs. 47% ± 11% with placebo) and area of no-reflow (14% ± 14% of infarct size with diazoxide pretreatment vs. 46% ± 20% with placebo). With diazoxide from 50 to 60 min coronary occlusion, however, there was marked hypotension, and infarct size (44% ± 7%) and area of no-reflow were not reduced (35% ± 25%).

**Conclusions:**

Cardioprotection by diazoxide pretreatment was confirmed in adult pigs with reperfused acute myocardial infarction but is not feasible when diazoxide is administered in a more realistic scenario before reperfusion and causes hypotension.

## Introduction

Cardioprotection is characterised by reduced infarct size and reduced coronary microvascular obstruction (1). Infarct size (2) and coronary microvascular obstruction (3) are major determinants of prognosis in patients with reperfused acute myocardial infarction. Despite a myriad of preclinical studies that demonstrated reduced infarct size and reduced coronary microvascular obstruction by mechanical or pharmacological cardioprotective interventions, the translation of cardioprotection to patient benefit has been largely disappointing so far (4–6). The lack of translation has been attributed to two major factors: (1) The recruitment of low-risk patients into cardioprotection trials in whom it was difficult to show a further reduction in mortality and heart failure development (7, 8); (2) The typical presence of advanced age, comorbidities, and co-medications in patients which were, however, not present in most preclinical studies but interfere with the signal transduction of cardioprotection (9). The signal transduction of cardioprotection by ischaemic conditioning and many pharmacological agents comprises an action on the sarcolemma and its channels and receptors, an activation of cytosolic protein kinase cascades, and ultimately an action on mitochondria and their function (10). To reduce an interference of comorbidities and co-medications with cardioprotective signal transduction to a minimum, it appears prudent to target its most distal signalling step, i.e., the mitochondria. Many mitochondrial ion channels have been targeted successfully in preclinical studies, and this action resulted in cardioprotection (11). However, phase III clinical trials targeting mitochondrial sites such as the permeability transition pore with cyclosporine A (12, 13) or other less defined sites (14) have not been successful, despite promising proof-of-concept trials (15).

Diazoxide is a cardioprotective agent that targets the mitochondria, and it has not yet been clinically tested. Diazoxide was initially identified as an opener of mitochondrial potassium channels in isolated bovine cardiac mitochondria (16). It was first demonstrated to reduce cell death in isolated rabbit cardiomyocytes undergoing simulated ischaemia (17). Diazoxide reduced infarct size in isolated, saline-perfused rabbit hearts undergoing regional ischaemia/reperfusion (I/R), and the mechanism of action was related to opening of mitochondrial ATP-dependent potassium channels (K_ATP_) and the release of reactive oxygen species (ROS) (18). There appears to be also an action of diazoxide on the mitochondrial respiratory chain, which is independent of the activation of K_ATP_; in isolated rat heart mitochondria, diazoxide stimulates mitochondrial respiration and ROS formation at complex III but inhibits ROS formation at complex I during reverse electron transfer as it occurs during early reperfusion (19, 20). Also, diazoxide interacts with Connexin 43 to release ROS (21, 22). These ROS that were released from the mitochondria in response to diazoxide must be considered as cardioprotective signals. These ROS might act to oxidise protein kinases that are then not only upstream of mitochondria in the cardioprotective signal transduction but also downstream (23). In fact, diazoxide might signal through oxidation of a protein tyrosine kinase since its cardioprotective effect is abrogated with genistein (18). Oxidation of protein kinase A—not, however, in response to diazoxide—has been demonstrated to reduce infarct size by prevention of lysosomal loss of calcium into the cytosol and detrimental calcium overload (24). Also, the mitochondrial ROS downregulates L-type calcium channels, thus possibly contributing to attenuated calcium overload (25). Diazoxide’s cardioprotective actions are antagonised by the non-selective K_ATP_ antagonist glibenclamide (18, 26) and the mitochondrial-selective K_ATP_ antagonist 5-hydroxydecanoate (18, 22). The cardioprotective properties of diazoxide were then related to a temporally and spatially restricted ROS release from mitochondria and in close interaction with mitochondrial Connexin 43. Diazoxide has also demonstrated cardioprotective properties in large animal models. Pretreatment with diazoxide reduced infarct size in sexually immature Yorkshire pigs (27) and creatine kinase-MB release in female piglets (28). Also, the addition of diazoxide to a cardioplegic solution attenuated stunning in pig hearts following global I/R (29, 30). However, in adult pigs when regional ischaemia was followed by cardioplegic arrest before reperfusion, diazoxide decreased infarct size in one study when added to cardioplegia (31) but not in another study with pretreatment of diazoxide before regional ischaemia (32); thus, the protection by diazoxide does not appear to be a robust finding. Also, diazoxide acts on vascular smooth muscle cells and induces profound vasodilation. When given systemically, diazoxide markedly reduces blood pressure, and this clinical indication is approved. A marked hypotension below the coronary autoregulatory range would possibly be counterproductive and promote ischaemia (33), when diazoxide is given systemically in a clinically realistic scenario of cardioprotection, i.e., during coronary occlusion and before reperfusion. The present study, therefore, attempted to: (1) confirm the cardioprotective effect of diazoxide pretreatment in sexually mature minipigs at stable haemodynamic conditions; such confirmation is not trivial since, e.g., infarct size reduction with ischaemic preconditioning was effective in Göttingen minipigs but not in Ossabaw minipigs (34); and (2) to see whether diazoxide would still exert cardioprotection when given just before reperfusion and when it is associated with profound hypotension.

## Methods

Unless otherwise specified, materials were obtained from Sigma-Aldrich (Deisenhofen, Germany).

The experimental protocols were approved by the Bioethical Committee of the district of Düsseldorf (G1868/21) and conform to the guidelines from Directive 2010/63/EU of the European Parliament on the protection of animals used for scientific purposes. We followed the ARRIVE 2.0 guidelines (35, 36) and the AVMA guidelines. A full description of the intended experimental design and analysis has not been published in a preclinical registry. Experiments in pigs were performed between June 2022 and February 2023.

### Experimental preparation

Validity of animal species and model selection: This *in vivo* experimental model in pigs replicates many aspects of the human reperfused myocardial infarction, notably its temporal and spatial development (37). Female Göttingen minipigs were purchased from Ellegaard, Dalmose, Denmark. We have recently demonstrated that infarct size and cardioprotection by ischaemic preconditioning are not sex-dependent in our model (38). Pigs were fed with standard chow (twice 300 g/day, #V4133. ssniff, Soest, Germany), had access to water *ad libitum*, and were kept in tiled rooms (∼2 m^2^/pig) with straw-bedding at 12 h/12 h light/dark cycles. There was a daily visual inspection of pigs’ health by animal caretakers and veterinarians. All behavioural abnormalities were recorded and monitored; only inconspicuous, apparently healthy pigs were included in the experiment. Pigs were 17 ± 5 months and weighed 36 ± 9 kg. Pigs were sedated with flunitrazepam (i.m.: 0.4 mg kg^−1^). Anaesthesia was induced with etomidate (i.v.: 0.3 mg kg^−1^, Hypnomidate, Voorschoten, The Netherlands) and sufentanil (i.v.: 1 µg kg^−1^, Sufentanil-hameln, Hameln, Germany). Anaesthesia was maintained with isoflurane (2%, TEVA, Eastbourne, United Kingdom) during artificial ventilation with room air (tidal volume: 8–10 ml kg^−1^, respiratory rate: 10–16 breaths min^−1^, inspiratory peak pressure 18–25 cm H_2_O, positive end-expiratory pressure: 5–7 cm H_2_O). Muscle relaxation during electrosurgery was induced with a single bolus of rocuronium (i.v.: 0.6 mg kg^−1^, B. Braun, Melsungen, Germany). This anaesthetic regimen is identical to that used in our institution for patients undergoing surgical coronary revascularisation (39). The pigs were placed on a heated table and covered with heated blankets to keep the oesophageal temperature between 36.0 and 38.0°C. ECG-lead II was continuously recorded using a single-channel, calibrated amplifier. A midline cervical incision was performed. The left jugular vein was cannulated for volume replacement and intravenous drug administration, and the right common carotid artery was cannulated to measure arterial pressure. The heart was exposed by a left lateral thoracotomy and instrumented with a micromanometer (DPT-6000, Codan-PVB, Forsting, Germany) in the left ventricle to measure left ventricular pressure (LVP; LabChart 8, Lab Chart, AD Instruments Pty LTD, New South Wales, Australia) and a Teflon catheter in the left atrium for the injection of coloured microspheres (40). Atrial pacing electrodes were sutured to the right atrium for eventual pacing with pulses of 1 V amplitude and 0.002 s duration (LabChart 8). The distal aortic arch was cannulated to withdraw the reference sample for regional blood flow measurement. The left anterior descending coronary artery (LAD) was dissected and prepared distal to its second diagonal branch for later coronary occlusion. The proximal descending aorta was equipped with a ribbon (“Bühner-Band”, Covetus GmbH, Düsseldorf, Germany), which could be manually tightened to maintain LVP.

#### Regional myocardial blood flow

Coloured microspheres were recovered from transmural myocardial samples taken from the central area at risk by digestion with 4 mol L^−1^ KOH and subsequent filtration (8 µm pore size, Pieper Filter, Bad Zwischenahn, Germany). Fluorescent dye was resolved from microspheres and quantified in a spectrophotometer (F-7100, Hitachi High-Tech, Krefeld, Germany). Blood flow was calculated as blood flow per tissue mass.

#### Area at risk, infarct size, and no-reflow

Thirty millilitres of warm 4% thioflavin-S solution (Morphisto, Frankfurt, Germany) was filtered through a 0.2 µm syringe-filter to remove particulate debris and slowly infused into the left atrium to demarcate non-perfused areas of the left ventricle after 180 min reperfusion (41, 42). Thereafter, the LAD was re-occluded at the same location as for the index ischaemia, and 5 ml blue dye (Patentblau V, Guerbet GmbH, Sulzbach, Germany) was quickly injected into the left atrium to delineate the area at risk as remaining unstained. The heart was quickly removed from the chest, rinsed with cold saline, and cut into five slices perpendicular to the ventricular long axis. The tissue slices were examined under ultraviolet light (340–360 nm, VL-UVA 135.11, Vilber Louramat, Eberhardzell, Germany). Areas without yellow-green fluorescence (thioflavin-S-negative) were encircled by incisions. After documenting the slices using a digital camera, the slice shape, the thioflavin-S-negative areas, and the demarcated area at risk were transferred to a transparent film. Thereafter, infarcted tissue was demarcated by triphenyl tetrazolium chloride (TTC) staining (1% dissolved in 90 mmol L^−1^ sodium phosphate buffer containing 8% dextran, Roth, Karlsruhe, Germany). The TTC-stained slices were again photographed, and transferred together with the tissue areas that remained unstained by TTC to the same transparent film that was used to document the area at risk and the no-reflow areas. Particular care was taken to proper realign the slices using “landmarks,” such as the position of papillary muscles and the incisions surrounding the no-reflow areas.

The transparent films were scanned and analysed using digital planimetry. The following areas were calculated and averaged for both sides of each slice: total area of the left ventricle, the area at risk, the area of TTC-negative tissue (infarcted), and the area of thioflavin-S-negative tissue within the infarcted tissue (no-reflow). Using the slice weight for normalisation, the tissue masses for the area at risk, the no-reflow area, and for the infarcted area were calculated. In addition, the area at risk was calculated as a fraction of the left ventricle, infarct size was calculated as a fraction of the area at risk, and the area of no-reflow was calculated as a fraction of infarct size.

### Protocols

Pigs were randomised (using sealed envelopes) to an I/R diazoxide pretreatment or diazoxide before reperfusion protocol, respectively.

#### Ischaemia/reperfusion

When the heart rate at baseline was <95 min^−1^, atrial pacing was performed. After stabilisation for at least 30 min, systemic haemodynamics and regional myocardial blood flow were measured. After intravenous infusion of unfractionated heparin (2,500 IU, Heparin-Natrium-ratiopharm, Ulm, Germany), the LAD was occluded distal to its second diagonal branch using a microvascular clamp (TKL-1, BIOVER^R^, Hergiswil, Switzerland). Heparin (2,500 IU) was again given at 25 and 55 min coronary occlusion. At 5 and 55 min coronary occlusion, systemic haemodynamics and regional myocardial blood flow were measured again. Reperfusion was induced after 60 min coronary occlusion by quick removal of the vascular clamp and visually confirmed by the reappearance of red colour on the surface of the reperfused myocardium. Systemic haemodynamics were again measured at 10 and 180 min reperfusion and regional myocardial blood flow at 10 and 180 min reperfusion. Ventricular fibrillation during ischaemia or reperfusion, as identified from the continuous lead II ECG recording, was immediately terminated by intra-thoracic defibrillation (up to 50 Ws; 6/4 ms biphasic pulse; Zoll R Series Monitor & Defibrillator, Zoll Medical Cooperation, Chelmsford, MA, United States). We did not use any antiarrhythmic or inotropic agents during resuscitation since they might interfere with the infarction process and/or cardioprotection. At the end of the experiment, pigs were euthanised by intracardiac injection of 20 ml potassium chloride (1 mol L^−1^).

#### Diazoxide pretreatment + I/R

The experimental protocol was identical to that of I/R, except that diazoxide (7 mg kg^−1^ i.v. over 10 min in the solvent solution of 10 ml NaCl, B. Braun with 20% 1 mol L^−1^ NaOH, pH = 11.7) was administered after baseline measurements before ischaemia. The dose of diazoxide was determined with reference to prior studies (27, 28) and to preliminary experiments in our preparation. During the administration of diazoxide, LVP was maintained through adjustment of the aortic snare.

#### Diazoxide before reperfusion + I/R

The experimental protocol was identical to that of I/R, except that diazoxide (7 mg kg^−1^ i.v. over 10 min in solvent solution of 10 ml NaCl, B. Braun with 20% 1 mol L^−1^ NaOH, pH = 11.7) was administered after 50 min coronary occlusion over 10 min just before reperfusion. In this set of experiments, LVP was not adjusted with the aortic snare.

In the I/R experiments, intravenous 10 ml of the solvent solution over 10 min did not induce a haemodynamic response, and the infarct size data were well in line with our prior data in this model in the absence of such solvent solution (38).

### Data and statistical analysis

The investigator who quantitatively assessed haemodynamics, regional myocardial blood flow, infarct size, and area of no-reflow was blinded to the protocol. Explorative statistics were performed on all data to test for normal distribution (Shapiro–Wilk test) and to identify potential outliers. Data are presented as mean ± SD; individual data on infarct size and no-reflow are also presented as scatterplots. Statistical package SAS 9.4 (Cary, NC, United States) was used to analyse haemodynamic and regional myocardial blood flow data by two-way analysis of variance (protocol, time) for repeated measures. Data on area at risk, infarct size, area of no-reflow, and episodes with fibrillation/defibrillation were analysed by one-way analysis of variance. When analysis of variance indicated a significant main effect or interaction, Fisher’s least square difference (LSD) tests were used for comparison of single mean values. The data and statistical analysis comply with the recommendations on experimental design and analysis in pharmacology.

## Results

### Haemodynamics

At baseline, heart rate, LVP, regional myocardial blood flow, and area at risk were not different between the three groups of I/R without or with diazoxide pretreatment or diazoxide just before reperfusion, respectively (Table 1). With the onset of ischaemia, regional myocardial blood flow was decreased markedly and not different between groups. LVP progressively decreased and heart rate increased. The intravenous administration of diazoxide during 50–60 min ischaemia aggravated the decrease in LVP pressure further, and this decrease persisted into early reperfusion. Regional myocardial blood flow during reperfusion recovered from I/R, displayed reactive hyperaemia in the group with diazoxide pretreatment, and remained depressed in the group with diazoxide before reperfusion. At the end of 180 min reperfusion, there were again no differences in heart rate and LVP between the three groups, but some increase in regional myocardial blood flow in the diazoxide groups persisted. There was no significant difference in ventricular fibrillation/defibrillation episodes between the three groups (Table 1).

**Table 1:** Area at risk, regional myocardial blood flow, heart rate, and left ventricular pressure in pigs undergoing ischaemia/reperfusion without and with diazoxide pretreatment or diazoxide before reperfusion.

	Ischaemia/reperfusion *n* = 5		Diazoxide pretreatment + ischaemia/reperfusion *n* = 5		Ischaemia + diazoxide before reperfusion *n* = 5		
		*p*-values vs. baseline		*p*-values vs. baseline		*p*-values vs. baseline	Two-way ANOVA results for RMBF, HR, and LVP and *p*-values for group comparisons
AAR (%LV)	25 ± 6		22 ± 6		22 ± 2		
Episodes with fibrillation/defibrillation	1 ± 1		4 ± 4		2 ± 1		
RMBF (ml/min/g)							
Baseline	0.91 ± 0.23		1.01 ± 0.23		1.14 ± 0.21		Main effects: protocol *p* = 0.0023 time: *p* < 0.0001 interaction protocol × time *p* = 0.0004
Diazoxide			2.58 ± 1.03	<0.0001			
15	0.06 ± 0.03	<0.0001	0.06 ± 0.03	<0.0001	0.05 ± 0.02	<0.0001	
155	0.04 ± 0.01	<0.0001	0.06 ± 0.03	<0.0001	0.02 ± 0.01	<0.0001	
R10	0.91 ± 0.34		2.18 ± 1.02	<0.0001	0.53 ± 0.20	<0.0078	<0.0001 diazoxide pretreatment vs. I/R and diazoxide before reperfusion
R180	0.87 ± 0.25		1.23 ± 0.43		0.89 ± 0.21		
HR (1/min)							
Baseline	101 ± 1		100 ± 0		96 ± 9		Main effects: protocol *p* = 0.0831 time: *p* = 0.6683 interaction protocol × time *p* = 0.7071
Diazoxide			100 ± 1		95 ± 10		
15	103 ± 5		99 ± 2		88 ± 22		
155	108 ± 27		107 ± 10		95 ± 22		
R10	108 ± 20		102 ± 7		109 ± 17		
R180	107 ± 14		103 ± 8		93 ± 9		
LVP (mmHg)							
Baseline	89 ± 7		92 ± 5				Main effects: protocol *p* = 0.0065 time: *p* < 0.0001 interaction protocol × time *p* = 0.0017
Diazoxide			89 ± 9				
15	81 ± 2	*0*.*0865*	81 ± 9	0.0237	85 ± 10	*0*.*0795*	
155	79 ± 8	0.0330	78 ± 14	0.0067	54 ± 10	<0.0001	<0.0001 diazoxide before reperfusion vs. diazoxide pretreatment and I/R
R10	73 ± 7	0.0010	79 ± 11	0.0085	62 ± 7	<0.0001	0.0007 diazoxide before reperfusion vs. diazoxide pretreatment 0.0283 diazoxide before reperfusion vs. I/R
R180	69 ± 7	<0.0001	78 ± 12	0.0067	73 ± 4	<0.0001	

AAR, area at risk; HR, heart rate; I5, 5 min ischaemia; I55, 55 min ischaemia; LV, left ventricle; LVP, left ventricular pressure; R10, 10 min reperfusion; R180, 180 min reperfusion; I/R, ischaemia/reperfusion; RMBF, regional myocardial blood flow.

Area at risk was analysed by one-way analysis of variance; haemodynamic and regional myocardial blood flow data were analysed by two-way analysis of variance (protocol, time) for repeated measures. Fisher's LSD tests were used to compare single mean values when analysis of variance detected significant effects.

### Infarct size and no-reflow area

**Figure 1:**
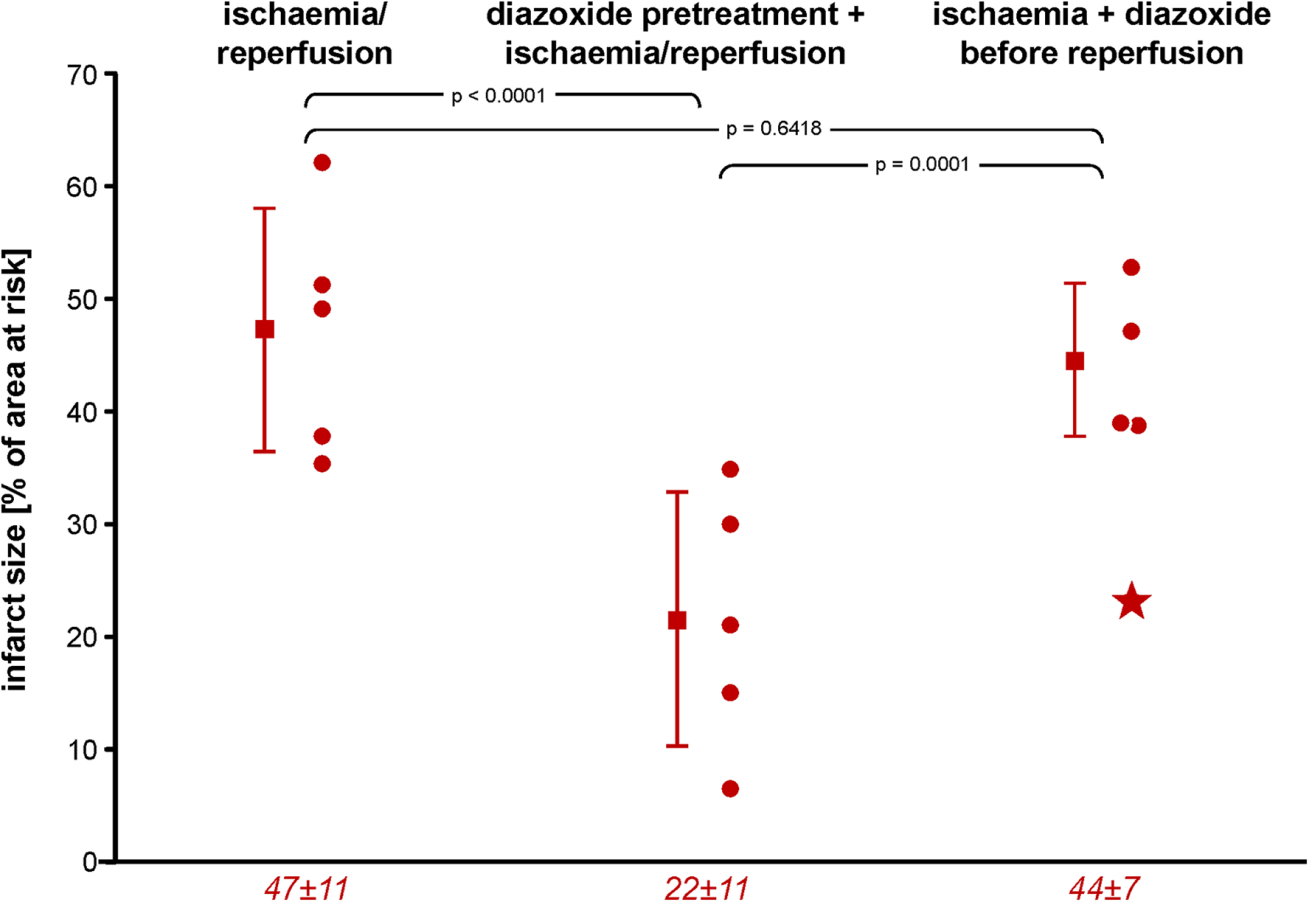
Infarct size following ischaemia/reperfusion without and with diazoxide pretreatment or diazoxide before reperfusion, respectively; individual data (circles) and means (squares) with SD; mean ± SD are also given as numerical data for ischaemia/reperfusion (*n* = 5), pretreatment with diazoxide + ischaemia/reperfusion (*n* = 5), and diazoxide before reperfusion (*n* = 4). The asterisk in the scatter plot of data with diazoxide before reperfusion indicates one pig with persistent TTC-positive staining within an area of no-reflow, as indicated by the negative thioflavin-S staining. Data were analysed by one-way analysis of variance and Fisher’s LSD *post-hoc test*s. LSD, least square difference; TTC, triphenyl tetrazolium chloride.

**Figure 2:**
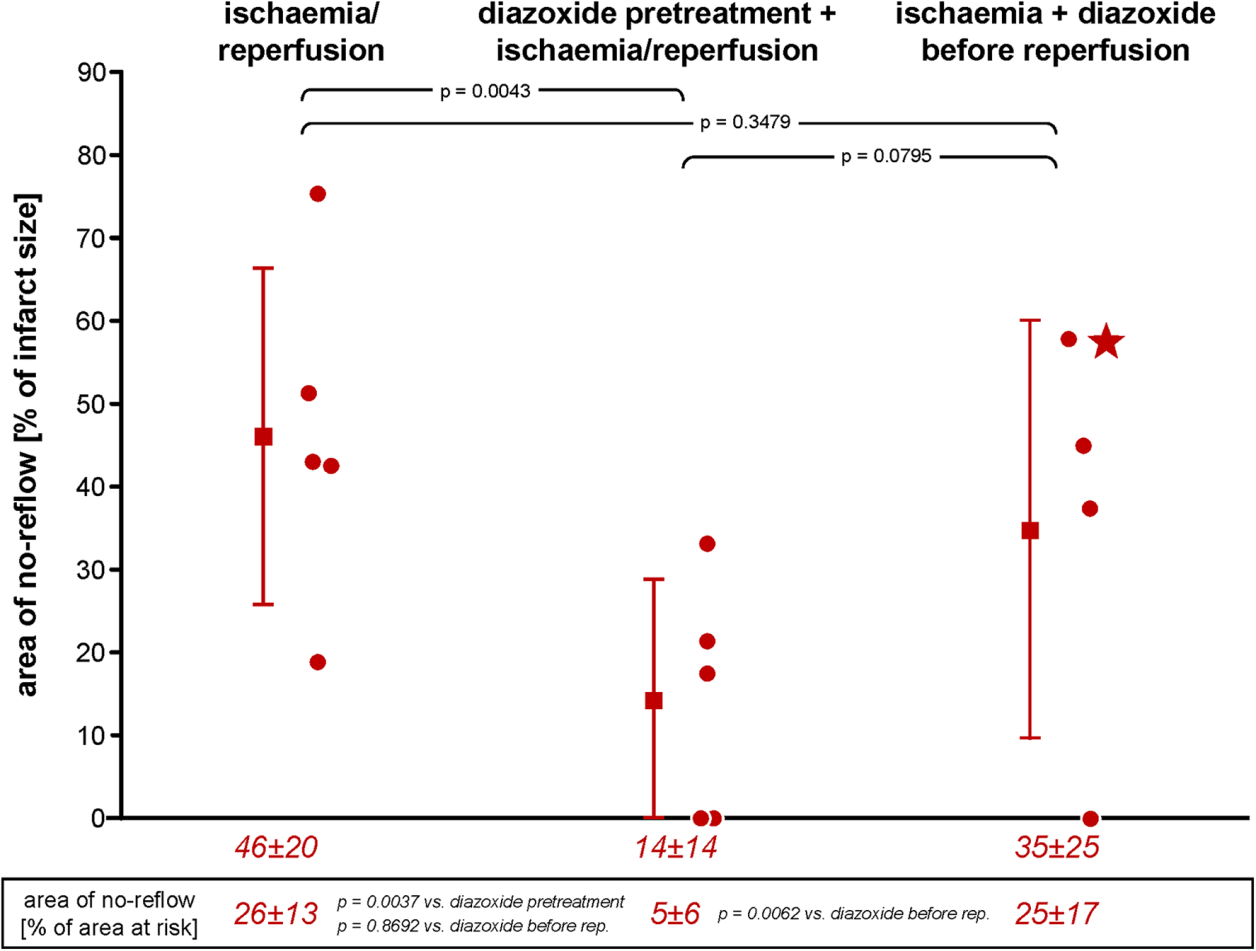
Area of no-reflow following ischaemia/reperfusion without and with diazoxide pretreatment or diazoxide before reperfusion, respectively; individual data (circles) and means (squares) with SD; mean ± SD are also given as numerical data for ischaemia/reperfusion (*n* = 5), pretreatment with diazoxide + ischaemia/reperfusion (*n* = 5), and diazoxide before reperfusion (*n* = 4). The asterisk in the scatter plot of data diazoxide before reperfusion indicates a pig with persistent TTC-positive staining within an area of no-reflow, as indicated by the negative thioflavin-S staining. Data were analysed by one-way analysis of variance and LSD *post-hoc test*s. LSD, least square difference; TTC, triphenyl tetrazolium chloride.

**Figure 3:**
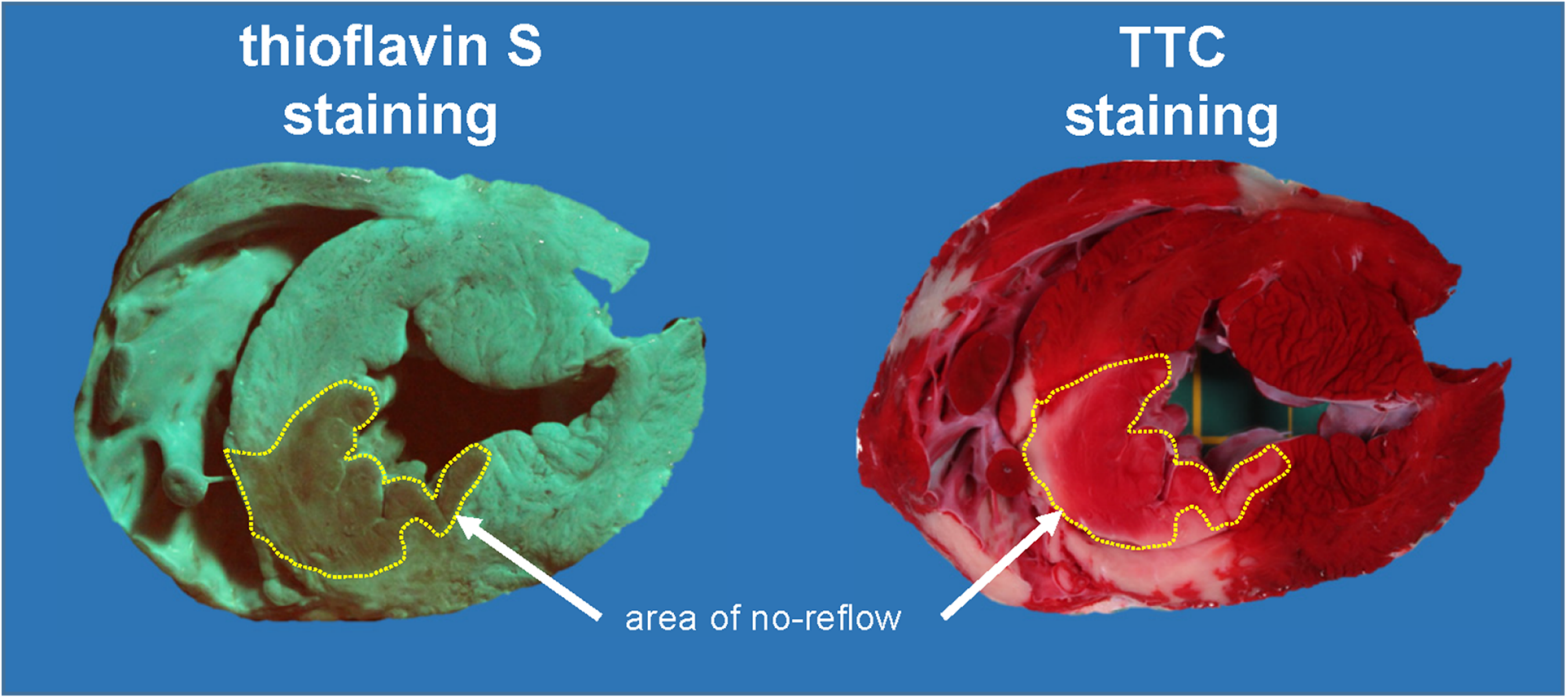
Original heart slice after demarcation of areas of no-reflow with thioflavin-S and after demarcation of avital tissue by TTC staining. The thioflavin-S-negative stained area (no-reflow) is indicated in both images. Almost the entire area of no-reflow is stained TTC positive. This pig had profound hypotension with intravenous diazoxide given over 10 min just before reperfusion (left ventricular pressure: 42 mmHg at 55 min ischaemia and 53 mmHg at 10 min reperfusion). TTC, triphenyl tetrazolium chloride.

Infarct size with I/R was 47% ± 11% of area at risk, and area of no-reflow was 46% ± 20% of infarct size; thus, both were very close to our prior data in this model (38). Diazoxide pretreatment with maintained haemodynamics (aortic snare) reduced both infarct size and area of no-reflow markedly (Figures 1, 2). However, when diazoxide was given systemically and induced hypotension, infarct size and area of no-reflow were no longer reduced. In one pig, there was marked hypotension into early reperfusion (LVP: 42 mmHg at 55 min ischaemia and 53 mmHg at 10 min reperfusion), associated with a large area of no-reflow, which resulted in the lack of TTC washout such that infarct size could no longer be clearly identified as TTC-negative (Figure 3).

## Discussion

The present study confirms the well-established fact that diazoxide is a powerful cardioprotective agent. In the present study, fibrillation/defibrillation episodes were not significantly different between control ischaemia/reperfusion, diazoxide pretreatment and diazoxide treatment at reperfusion, and if anything increased by diazoxide, thus excluding a primary antiarrhythmic action of diazoxide. However, diazoxide pretreatment before coronary occlusion and reperfusion and with maintenance of heart rate and LVP reduced infarct size, confirming in an adult pig model prior data from juvenile pig models of reperfused acute myocardial infarction (27, 28). Of note—and this is novel—cardioprotection was also seen in the coronary circulation (43), since the area of no-reflow was reduced by diazoxide pretreatment. Our study, however, did not only aim to confirm cardioprotection by diazoxide in adult rather than juvenile pigs and protection not only of cardiomyocytes but also of the coronary circulation, but our study also aimed for the translational potential of diazoxide. We, therefore, in a second approach, administered diazoxide in a more realistic scenario as would occur in a clinical setting of acute myocardial infarction, i.e., before reperfusion and without compensation of the marked hypotension that diazoxide induces upon systemic administration. Diazoxide before reperfusion and in the presence of marked hypotension, which added to the decrease in LVP inflicted by the coronary occlusion *per se*, no longer reduced infarct size and area of no-reflow. In fact, in a single extreme case, the loss of coronary perfusion pressure caused such pronounced microvascular obstruction that the washout of NADH oxidase and reducing NADH equivalents was delayed to an extent that TTC staining remained positive. These data clearly indicate that systemic administration of diazoxide before reperfusion could not induce cardioprotection, but in fact rather caused haemodynamic complications. Apart from the deleterious role of severe hypotension *per se*, the decrease in perfusion pressure and further decrease in regional myocardial blood flow will also have decreased the diazoxide delivery to the ischaemic myocardium. Theoretically then, one would attempt intracoronary administration of diazoxide before or at reperfusion. Unfortunately, however, diazoxide is poorly soluble (product information Sigma-Aldrich GCY/NSB 12/03) (44), requiring either a pH > 10 in saline (present study), high volume of ethanol or methanol, or use of toxic solvents such as dimethyl sulfoxide (45, 46) or dimethylformamide (47), and thus clearly precluding an intracoronary infusion of diazoxide into a reperfused myocardial region, which is anyway at jeopardy. Chronic systemic treatment with diazoxide would, apart from hypotension, is faced with further problems, in that its cardioprotective efficacy might be impaired by high cholesterol levels (26), and that itself through ROS-triggered ROS release from mitochondria and NADPH oxidase might induce nitrate tolerance (48–52).

**Figure 4:**
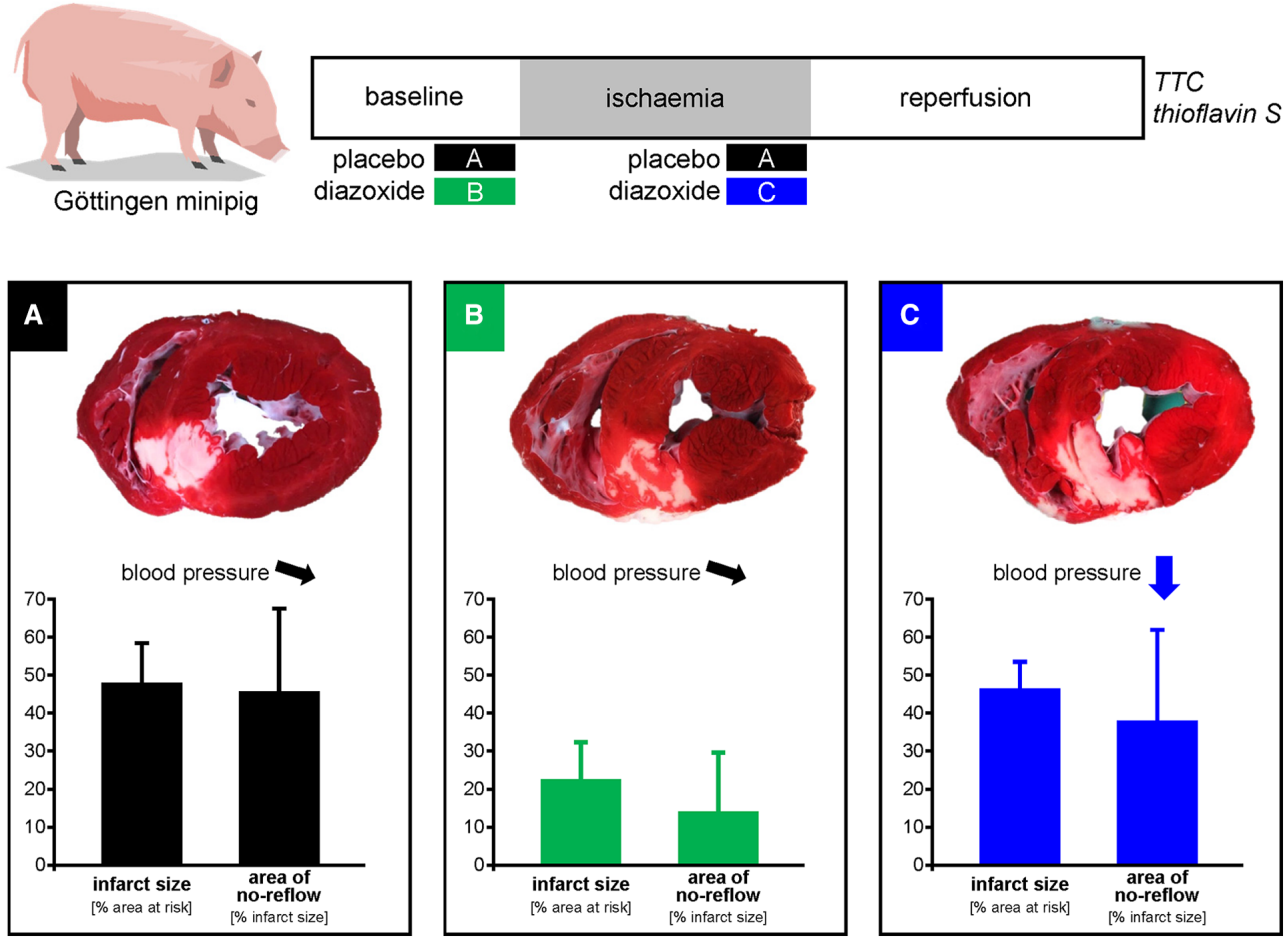
Schematic summary. Diazoxide activates mitochondrial ATP-dependent K-channels and stimulates respiration. Experiments in juvenile pigs had reported infarct size reduction by pretreatment with intravenous diazoxide. We now analysed the use of diazoxide in adult minipigs and in a more realistic scenario of administration before reperfusion. In comparison to placebo **(A)**, diazoxide pretreatment **(B)** reduced infarct size and area of no-reflow, but diazoxide before reperfusion **(C)** caused hypotension and did not reduce infarct size and no-reflow. Thus, cardioprotection with diazoxide is not feasible in a clinical scenario of acute myocardial infarction with administration before reperfusion.

We must, therefore, conclude that diazoxide is a powerful cardioprotectant with an action on mitochondrial K_ATP_ and respiration, but unfortunately it is not feasible for translation into a realistic clinical setting of acute myocardial infarction (Figure 4). Mechanistically, our study emphasises the pivotal importance of adequate coronary perfusion pressure above the autoregulatory range (33), which dominates during ischaemia and reperfusion above the potential protection by diazoxide. Gentle reperfusion over the first 30 min of reperfusion is protective, but perfusion pressure must be restored to above the lower limit of the autoregulatory range (53). Unfortunately, we did not measure aortic/coronary perfusion pressure in the present study. However, even LVP at 10 min reperfusion was still around 60 mmHg, and therefore coronary perfusion pressure was definitely less than 50 mmHg, which is the lower limit of coronary autoregulation in an anesthetised open-chest preparation. Our study also demonstrated that left ventricular unloading is not cardioprotective unless coronary perfusion pressure is preserved. Also, whereas gentle reperfusion may attenuate injury and reduce infarct size (53), perfusion pressure during such gentle reperfusion must still be above the lower limit of the autoregulatory range. Strategically, our study again emphasises that cardioprotective research must progress from reductionist models to realistic scenarios if it really aims for translation (54).

## Data Availability

The original contributions presented in the study are included in the article/Supplementary Material, further inquiries can be directed to the corresponding author.
